# The Interplay between Immunosenescence and Microbiota in the Efficacy of Vaccines

**DOI:** 10.3390/vaccines8040636

**Published:** 2020-11-02

**Authors:** Rossella Cianci, Laura Franza, Maria Grazia Massaro, Raffaele Borriello, Francesco De Vito, Giovanni Gambassi

**Affiliations:** 1 General Medicine, Catholic University of the Sacred Heart, Fondazione Policlinico Universitario A. Gemelli IRCCS, 00168 Roma, Italy; mg.massaro92@gmail.com (M.G.M.); raffaeleborr@gmail.com (R.B.); francesco.devito@policlinicogemelli.it (F.D.V.); Giovanni.Gambassi@unicatt.it (G.G.); 2 Emergency Medicine, Catholic University of the Sacred Heart, Fondazione Policlinico Universitario A. Gemelli IRCCS, 00168 Roma, Italy; cliodnaghfranza@gmail.com

**Keywords:** microbiota, vaccines, immunosenescence, inflammaging

## Abstract

Vaccinations are among the most effective medical procedures and have had an incredible impact on almost everyone’s life. One of the populations that can benefit the most from them are elderly people. Unfortunately, in this group, vaccines are less effective than in other groups, due to immunosenescence. The immune system ages like the whole body and becomes less effective in responding to infections and vaccinations. At the same time, immunosenescence also favors an inflammatory microenvironment, which is linked to many conditions typical of the geriatrics population. The microbiota is one of the key actors in modulating the immune response and, in this review, we discuss the current evidence on the role of microbiota in regulating the immune response to vaccines, particularly in elderly people.

## 1. Introduction

Vaccination is one of the most effective medical procedures and has had a significant impact on both quality of life and life expectation of people [1]. Yet, certain populations are at a higher risk of developing side effects from the administration of vaccines or not respond. Even though children are usually viewed as most at risk of developing such side effects, it is actually the elderly and the immune-suppressed who are at danger. The elderly population and persons who are immune-suppressed are also at a very high risk of developing potentially lethal infections, take longer to recover and often face long lasting sequelae.

Particular vaccination protocols have indeed been designed for these populations and live-vaccines are very rarely used, because of the risk of developing infections following the vaccination itself [2]. On the other hand, these populations have also a worse and less effective response to non-live-vaccinations: the use of immunity-boosters is mandatory to obtain any kind of response.

Immune-suppression in itself determines an altered response to vaccinations and elderly people often fall into this group, due mainly to malnutrition [3]. Yet, even those elderly persons who do not meet the requirements to be conventionally considered immune-suppressed, present an altered immune response, a condition known as immunosenescence [4]. Immunosenescence involves mainly the adaptive immune system, with a reduced ability to respond to new antigens, accumulation of memory T cells and the constant presence of low-grade inflammation, so called inflammaging. Furthermore, innate response undergoes some changes, particularly in terms of signal-transduction, but they are not as relevant [5]. Even though some of these changes could be partly explained by cellular senescence, there still is a lack of understanding of immunosenescence.

In recent years, the role of gut microbiota (GM) in modulating immunity has come under a lot of scrutiny. We will thus discuss the changes in immunity in the older population and the consensual changes in their microbiota. Furthermore, we will examine the role of microbiota in boosting the response to vaccinations.

## 2. Immunosenescence

Immunosenescence can be defined as the physiological age-associated changes of the immune system that determine an increased susceptibility to infectious pathogens and poor vaccine responses [6]. Immunosenescence, aggravated by co-morbidities, varies with age, becoming apparent after 60–65 years and more important after 85 years of age [7]. The underlying mechanisms though are not clear. Both qualitative and quantitative alterations concerning innate and adaptive immunity have been observed. Furthermore, in older adults a state of a systemic chronic low-grade inflammation, defined inflammaging by some authors, can be observed and seemingly contributes to the dysregulation of the immune systems [8]. Inflammaging can be influenced by many factors such as environmental and metabolic factors, individual’s diet, nutrition and gut microbiota [9].

## 3. Innate Immune Response

Regarding the alterations observed in innate immune response, with advanced age, neutrophils reduce their ability to migrate to infection sites. This has been mainly linked to the development of signal transduction defects. Specifically, a fundamental p1athway is the phosphatidylinositol-3 kinase (PI3K) pathway which is normally activated by chemokines through mediated protein G receptors present on neutrophil membranes; this binding results in the phosphorylation of phosphatidylinositol 4,5-bisphosphate. The aberrant activation of this signaling cascade would cause altered neutrophil migration to the infection site [10]. Furthermore, the activity of phagocytosis also appeared to be reduced both because of the reduced expression of the Fcγ receptor CD16 [11,12] and the lower production capacity of reactive oxygen species [13].

There are also changes in the activity of macrophages: in advanced age, a reduction in the production capacity of cytokines (mostly IL6 and TNF alpha) is observable, probably due to an altered expression of Toll like receptors (TLR) [14]. The mechanisms underlying this altered production are complex and not entirely clear [9]. At the same time, with aging, macrophages develop a defect in macro-autophagy [15]. This defect causes an accumulation of macrophages and consequentially inflammatory cytokines, which contributes to the previously mentioned inflammaging [9]. Macrophages reduce the ability to respond to IFN-γ with a simultaneous reduction in the phosphorylation of the STAT-1alfa pathway [16]. This pathway is fundamental for macrophage activation and for IFN-dependent production of superoxide anion. Finally, monocytes and macrophages appear to express lower levels of HLA and MHC class II with age on their surface [17,18].

Age-associated alterations in Natural Killer (NK) cells consist of a progressive reduction of expression of the CD56^bright^ receptor, which has a mainly immunoregulatory function, with a simultaneous increase in NK cells expressing the CD56^dim^ receptor, that provides cytotoxic actions. This results in a reduced responsiveness to cytokine signaling [19]. The remaining CD56^bright^ cells develop a greater capacity to the response to INF-γ but this phenomenon has not been noticed for the other cytokines [20,21].

During immunosenescence a defect in the connection between innate and adaptive immune responses occurs. Plasmacytoid dendritic cells and myeloid dendritic cells in older adults reduce their ability to present antigen and to stimulate CD4^+^ and CD8^+^ T-cell activation [22]. Moreover, follicular dendritic cells develop an age-related reduction in Fcγ RII receptor expression which causes a defect in the formation of germination centers. This causes an overall alteration in B-cell proliferation and antibody production [23].

## 4. Adaptive Immune Response

Concerning adaptive immunity system, many alterations have been observed and are probably central in the development of immunosenescence and possibly in the deficient response to vaccines in the elderly population.

B cells play a pivotal role in the humoral component of the adaptive immune system, secreting antibodies, with their activity of antigen-presenting and secreting cytokines. With aging, maturation of B cells in bone marrow is impaired due to a reduced production by stromal cells of IL-7, which is an important growth factor for maturing B cells; B cell progenitors also appear to be less responsive to IL-7 [24]. Studies conducted in mice linked this impairment with a state of chronic inflammation in the bone marrow [25]. However, human studies about this defect are limited. Interestingly, a reduced serum level of B-cell activating factor (BAFF) has been found in studies conducted in humans. BAFF is an important factor connected to the survival of B-cells [26]. However, the total number of B cells remains stable with age [27], which suggests that there is a reduced cellular turnover with a simultaneous accumulation of aged B cells that present defects in normal functions, such as the ability to recognize and respond to new antigens. Specifically, aged B cells have a reduced diversity of B-cell receptor (BCR) [28]. An intrinsic defect in class-switch recombination also contributes to the reduced responsiveness to new antigens. This defect seems to be related to the altered transcription of the E47 factor, with a consequent dysregulation in the expression of the activation-induced cytidine deaminase (AID), essential for the recombination process [29]. Furthermore, aged B cells show a reduced ability in differentiating into plasma cells and therefore in the ability to produce antibodies [30], and at the same time a spontaneous and unmotivated production of TNF-α can also be observed and contributes to the previously mentioned inflammaging [31].

**Figure 1:**
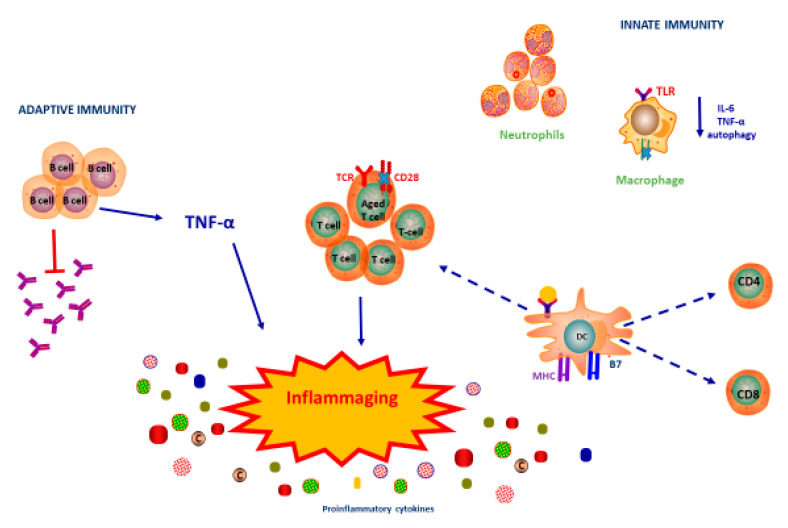
**Immunosenescence and inflammaging.** Several factors are implicated in immunosenescence. Neutrophils reduce their ability to migrate to infection sites. Macrophages show a reduction in the production capacity of cytokines. Dendritic Cells (DC) reduce their ability to present antigen and to stimulate CD4+ and CD8+ T-cell activation. B cells have an intrinsic defect in class-switch recombination and present lower production of antibodies and higher production of TNF-α that contributes to inflammaging. TNF-α inhibits the transcription of CD28. Furthermore, aged T cells show a higher secretory activity contributing to inflammaging.

Regarding T cells, similarly to B cells, their total number does not vary with age [32]. However, there are intrinsic changes in T cells, which modify the cellular immune response. The physiological defect of production of cytokines and growth factors by thymic cells, caused by thymic involution, determines a reduction in circulating naive T cells [33]. It has been hypothesized that chronic infective states play a role in the altered immune response mediated by CD8 + T cells [9]. This has been supposed observing results from studies in older adults with CMV infection. Specifically, it was observed that chronic CMV infection causes oligoclonal expansion of CMV-specific memory CD8 + T cells; this causes a reduction in the CD8 + T cell repertoire in the periphery capable of responding to other antigenic stimuli [34]. In addition, aged T cells reduce their expression of the costimulatory regulator CD28, which is fundamental for a complete activation of T cells [35], with a not entirely clear mechanism; TNF-α seems to play a role in this defect, as it is able to inhibit the transcription of CD28 [36]. Furthermore, studies conducted in mice and humans show an altered production of cytokines in T cells. More specifically, effector memory cells have a reduced production of cytokines in response to the antigen [37,38], while terminally differentiated senescent CD4 + T cells show a higher secretory activity contributing to the already mentioned inflammaging [39]. In conclusion, immunosenescence can be defined as the result of alterations in the function of all the branches of the human immune response, which causes a defect in the normal homeostasis of the immune system, resulting probably in a major susceptibility of infections and a poorer response to vaccinations (Figure 1).

## 5. Immunosenescence and Inflammaging

As mentioned previously, inflammaging refers to high self-reactivity in the elderly, resulting in a typical chronic, low-grade systemic inflammation, mainly driven by adaptive immunity, particularly altered T-lymphocytes, as discussed below [40]. Inflammaging is closely associated to aging, thus it can be, to some extent, considered as the other side of immunosenescence; indeed, the two processes are closely linked [41].

Specifically, the increased number of memory T lymphocytes and that of B lymphocytes typical of old age, might be caused by the continuous chronic antigenic stimulation, mimicking inflammaging. A consequence of chronic stimulation is exhaustion, characterized by the emergence of inhibitory receptors, such as PD-1, CTLA-4 and others, which impair the immune system function [42]. These alterations can cause an impairment of normal immune function, which exposes the elderly patient to an increased risk of infections, cancer and chronic diseases [43].

The thymus also plays an important role in inflammaging. With age, indeed, thymus atrophy decreases its ability to establish a central tolerance, thereby, causing an increase in the number of circulating self-reactive T cells, thus increasing overall inflammation. Normally, Treg cells suppress self-reactivity, yet, aged Treg cells are usually unable to do this [44]. Indeed, the alteration in controlling autoimmunity that takes place in the elderly has also a negative impact in terms of safety of vaccinations in this population, even though at present data seems to suggest that it is still an acceptable risk [45]. Overall, the altered function of adaptive immunity appears to be the main driving force behind inflammaging.

## 6. Immunosenescence in the Context of Reduced Vaccines Response

The multiple phenomena observed in elderlies’ immune system and described above can be considered responsible of the demonstrated poorer immunological response of vaccination in older adults [46,47]. Considering the increased severity of viral and bacterial infections, and the higher risk of acute and long-term sequelae [48], suboptimal response to vaccination of older adults is an emerging public-health problem, which has led to the consciousness that specific strategies for this population are needed. Today we assist to a real new impulse in researching possible ways of boosting elderlies’ response to vaccines, which has already led to experimenting alternative strategies to overcome this problem, e.g., the use of new influenza high-dose and adjuvated vaccines in this population [49].

The association between age-related changes in regulation of immune response and vaccine response are supported by several observation, even if a mechanistic explanation of the phenomenon is still lacking. Most cellular alterations in the elderlies have been observed in studies concerning the response to the influenza vaccine and are discussed below.

First of all, the poorer immune response to influenza vaccine in elderlies is well known, with an estimated proportion of seroconversion of only 10–30%, compared to 50–75% in younger individuals [50,51]. Furthermore, even when reaching seroconversion, older adults develop a less varied antibody repertoire against the virus [52]. In the context of cellular immunity, studies in mice show that in aged subjects CD8+ T cells exhibit limited diversity in their TCR than in younger individuals [53]; furthermore, a reduction in Th1 cells producing inflammatory cytokines has been noted [54]. An observation obtained in humans shows a different pattern of clonal expansions in effector T cells after influenza vaccine, with a major expansion of CD45RA+CD28-CD8+ T cells in older adults; this has been implied by some authors to contribute in an impairment in Th1/Th2 cytokine production, resulting in a limitation in efficacy of the antibody response [55]. Differences in the response of NK cells have also been observed. A study shows that between older adults and younger individuals there are different modifications in NK cells subtypes after receiving influenza vaccine: moreover, in elderlies vaccination significantly decreases the proportion of CD3-CD56+ and CD3-CD56+CD57+ NK cells, but not in younger individuals. In contrast, after vaccination, younger subjects present a greater number of CD56^bright^ cells and a lower of CD56^dim^ cells [56].

Other evidence supporting the link between immunosenescence in older adults and poorer vaccine response are available from studies conducted in mice and humans who received vaccine for pneumococcal disease. Indeed, studies in mice show an impairment in IgA production after nasal vaccination with aging [57,58]. In humans, antibodies from older adults have diminished opsonization activity against S. pneumoniae compared to younger individuals [59,60].

In conclusion, immunosenescence in older adults manifests itself also through to a poorer response to many of the most recommended vaccines for this population, probably representing the biological base of this phenomenon. Understanding the mechanisms of aging of immune system is a fundamental step to improve older adults’ healthcare and guarantee better strategies to reduce the risks that come from the most common infectious diseases.

## 7. Immunity and Microbiota

An interesting role in the changes of the immune system of the elderly population may be explained by the changes occurring in the gut microbiome (GM) in this group. GM has emerged in recent years as an important immune modulator: its composition plays a preponderant role in many diseases, from cancer to autoimmune pathologies, and determines overall health in each individual. Even diseases such as Parkinson’s and Alzheimer’s are influenced by signals sent by the microbial population living in our intestine, to such an extent that it is possible to talk about a gut-brain axis, intensely modulated by one’s GM. On the other hand, GM also undergoes massive changes, following diet and drug consumption, which in turn are highly influenced by GM composition.

The way by which GM could alter immune system in the host and vaccination response is still unknown and object of many observational and interventional studies. Even if the discussed interplay involving many classes of molecules and cellular interactions is at least in part responsible, the growing attention toward the class of molecules of the Short-Chain Fatty Acids (SCFAs), produced by several species in GM, is remarkable [10]. Overall, although the mechanisms are not exactly known, a role of gut microbiota in vaccination response is recognized in medical literature [61]. According to several studies conducted in recent years [62,63,64,65], there is a correlation between the composition of the microbiota and the individual’s response to oral and parenteral vaccines. According to a recent review [66], it appears that children with an abundance of the phylum Actinobacteria in their GM have a higher humoral and cellular response to some oral and parenteral vaccines [62,65]. Likewise, a higher expression of Proteobacteria is associated with a lower cellular and humoral response [62]. In both children and adults, a high prevalence of the phylum Firmicutes is associated with high cellular and humoral response to oral vaccines [63]; a higher prevalence of phylum Bacteroidetes in children is associated with a lower humoral response to oral vaccines [64].

Other molecules observed having a role in this interaction are the peptidoglycan component muramyl dipeptide (MDP) and the nucleotide binding oligomerization agonists containing domain 2 (NOD2), that also have been observed having an adjuvant effect for the cholera vaccine administered via nasal cavity in a study conducted in mice [61,67]. Antibiotic treatment in these mice determined the suppression of the humoral response to the vaccine and the consequent immunization of the mucosa; on the contrary, the reconstruction of the intestinal microbiota with a NOD2 agonist determined a good vaccination response [67]. This confirms that GM influences the mucosal adjuvant activity [61].

The production of LPS endotoxin by several bacteria present in the microbiota could influence the antibody response to the vaccine [68], although it is not yet known how. LPS has indeed an immunomodulatory activity that can be important, especially when the vaccine is administered after an antibiotic therapy which normally causes an overgrowth of bacteria, including Enterobacteriaceae, which produce high levels of LPS [69].

Furthermore, probiotics, alive microorganisms, with a potentially useful effect for the host when administered orally, have been analyzed in the response to vaccines [70]. A recent review [71] of 26 studies conducted on 17 different types of both oral and parenteral vaccines, documented the beneficial effect of probiotics in vaccination response in about half of the studies analyzed. This effect appears stronger for oral vaccinations and parenteral influenza vaccination. A subsequent review conducted on four studies about oral vaccines [72] did not confirm the beneficial effect of probiotics.

Cho et al. conducted a study using a middle-aged mouse model to demonstrate the close link between immunosenescence and the disruption of the gut microbiota that occurs in old age. Treatment with high-dose syringaresinol (SYR), a polyphenolic chemical isolated from Panax ginseng berry pulp, delayed age-related changes in naïve T lymphocytes and Treg cell populations and reduced inflammation in mouse models. The treatment also significantly improved the composition of the microbiota, particularly favoring *Lactobacillus* and *Bifidobacterium* bacteria populations, reducing opportunistic pathogens. Treatment also improved humoral response to influenza vaccination at the level of young healthy controls [73].

This complex network of interactions between GM and immune response in the host must be considered in the wider context of impaired immune response in older adults. The amount of cellular and molecular alterations previously mentioned and synthesized by the concept of immunosenescence, could be indeed a consequence or even a causal factor for alterations in normal GM equilibrium and composition with aging. In fact, it is well known that GM is susceptible of changing because of many acquired factors, the most important of which are diet, health status, drugs intake and lifestyle [61,62]. These changes mostly occur in older adults, for quite intuitive reasons such as following a very poor diet, the high drug consumption, and the number of comorbidities. Thus, it has been observed that gut microbiome is quite different among elderly and younger subjects [63].

Response to vaccination is extremely variable: age, health status, host genetics, nutritional status and vaccine composition are all factors that need to be taken into consideration. Immunological imprinting following as a result of prior exposure to the pathogen and the prevalence of chronic infections such as tuberculosis, HIV or parasites may also have an impact [74]. Elderly patients furthermore have significantly lower response rates. However, the administration of multiple immunogenic vaccines helped to increase response rates and improve the overall efficacy of vaccinations [44].

A new chapter that is emerging is the role of GM in modulating immune response in general and towards vaccination, in particular. GM modulates immunity in many ways and not only at a local level. Certain bacteria are well known promoters of inflammation (e.g., *E. faecalis*, *C. septicum*), while other bacteria have an anti-inflammatory effect, particularly the SCFAs-producers. GM appears to be involved in a complex cross-talk with various components of the immune system, both in its innate and adaptive components. T-lymphocytes, for instance, are influenced in their differentiation by the different microbial populations living in the gut: *B. fragilis* is capable to influence the development of T-regs, with an overall anti-inflammatory effect, while its Enterotoxigenic variant promotes the differentiation of Th17 lymphocytes, which seem to promote tumorigenesis in mice [75]. Furthermore, GM can have a direct barrier effect on the intestine: the presence of certain bacteria avoids the growth of other species and can prevent the absorption of certain nutrients.

Orally administered vaccines have been studied in relation to microbiota composition and the results have proven that different GM composition does influence the response to vaccination. A lower socio-economic status and a poor diet, for instance, have been linked to a poor response to vaccination. This has emerged both for polio and for rotavirus oral vaccination [76,77,78].

It is important to note that the altered response in this population to orally formulated vaccinations is particularly unfortunate, as these groups of the population are the ones that would mostly benefit from oral vaccination, yet it is not surprising that a poor nutritional status has a negative impact on vaccine-response. As shown by Arrietta et al. [79], the early exposure to fecal bacteria has dire consequences on the development of children, leading to the development of enteritis which in turn determines a chronic malnutrition status, one of the causes of immune-deficiency. Children living in poor areas are far more at risk of a similar exposure, also given the poor hygienic conditions and, thus, are far more likely to develop nutritional immune deficiency. Furthermore, maternal nutritional status plays an important role in determining the composition of GM in children and this further increases the risk of this group of developing severe dysbiosis and all the associated consequences [80]. Another aspect that needs to be considered is the fact that, based solely on geography, the GM varies quite a lot. As most vaccines are designed based on European and Northern American populations, this may influence the response rates to vaccinations of other populations.

The impact of GM in vaccine-response has been primarily studied in murine models. Germ-free did not develop a specific immune-response through B-lymphocyte differentiation, but once GM was restored, a TRL-5 response was activated and thus B-lymphocyte differentiation [81].

## 8. Microbiota in the Elderly

The above-mentioned studies all show the importance of early GM composition, but recent studies have concentrated on the importance of GM composition in the elderly, also in terms of response to vaccination.

**Figure 2:**
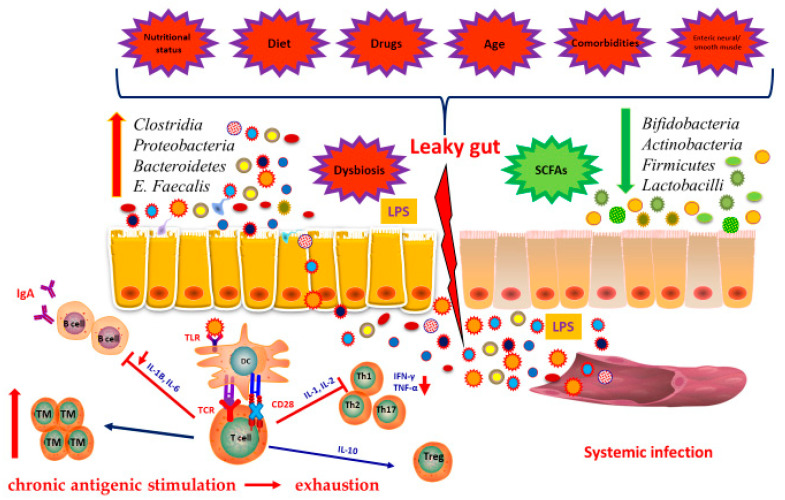
**Interplay between microbiota and immunity in elderly.** Changes in gut microbiota occur in the elderly. A condition known as “leaky gut” is associated to many diseases and common in the elderly. It is determined by alteration in several factors as diet, nutritional status, use of antibiotics or other drugs, the presence of comorbidities and the dysbiosis. Furthermore, due to leaky gut, gut microbiota can pass the gut barrier and enter the blood stream, a key step in the process of inflammaging. Furthermore, the continuous chronic antigenic stimulation causes the increased number of memory T cells. A consequence of chronic stimulation is exhaustion. Abbreviations: TM, memory T cells; DC, dendritic cells; LPS, lipopolysaccharide.

Microbiota in older persons tends to change when compared to the younger population, with an increased presence of potentially dangerous bacteria, such as Clostridia and Enterobacteria [82]. Yet, it still is not clear whether GM changes with age or whether it is a determinant of aging. What is clear is that healthy aging is influenced by the composition of GM: metabolic, neurologic and cardiovascular disease have all been linked to alterations in the composition of GM [83,84,85]. In some cases, though, what changes is not so much the composition if GM, but its capacity to pass the gut barrier and enter the blood stream, a key step in the process of inflammaging. Such phenomenon is known as “leaky gut”, a condition linked to many diseases and common in the elderly [86,87] (Figure 2). The increased permeability of the gut seems to be linked both to not modifiable changes (e.g., smooth enteric muscle changes, alterations of the enteric neural system) and life-style associated ones (e.g., drug consumption, diet changes) [88].

Elderly persons often follow a very poor diet, given changes in the hunger control centers in the brain and the high drug consumption and the composition of their GM is, thus, quite different from the one of the general population [89]. A diet rich in fats, such as the one of the Western World, may favor an “inflammatory” flora, which promotes extraintestinal disorders and diseases. A high-fiber diet instead promotes an “anti-inflammatory” flora: as it will be discussed further on, GM has not only local effects, but also systemic ones. Overall, it appears that persons 55 and older tend to have a worsening dietary pattern as time goes by [90].

Consumption of proton-pump-inhibitors (PPIs) is particularly dangerous in this population: by reducing the chloride production in the stomach, it favors bacterial overgrowth and is indeed known to be a risk factor in developing C. difficile infections in hospitalized patients [91].

Drugs in general affect the composition of the microbiota: antibiotics are the most obvious culprits and even short courses of antibiotic therapy can alter significantly the GM, promoting and aggravating diseases [92,93]. Non-steroid anti-inflammatory drugs (NSAIDs) and steroids can also modulate the composition of GM, partly because they are often prescribed with PPIs. Medications in general interact with GM in a bidirectional fashion: on the one hand their metabolism is influenced by the composition of GM and on the other hand they select GM.

Overall, some changes in the microbiota of elderly persons are so common that they can be considered a standard feature of the senior GM.

More specifically, the most common difference is the ratio between the different *gena* which compose the human microbiota: in adults Firmicutes are predominant, while in the elderly *Bacteroides* are dominant, Bifidobacteria and mucin-degrading *Akkermansia muciniphila* are also more common in the elderly than in the young. Yet, such changes are not present in those who live to be over one hundred, suggesting that the GM composition does impact longevity. In particular the Firmicutes in extremely old people are mostly represented by Bacilli, with *Clostridium IV* being far less common [94].

Overall, the most commonly present bacterial populations of the elderly seem to produce fewer Short-Chain Fatty Acids (SCFAs), which are essential in reducing inflammation, both through direct mechanisms (e.g., inhibiting the production of inflammatory mediators such as TNF-α, IL-6, and NO and by promoting IL-10) and indirect mechanisms (e.g., insulin response modulation, closely linked to metabolic syndrome; hormone modulation) [95,96,97] The reduction of such mechanism is indeed one of the hypothesized mechanisms underlying the process of inflammaging. Curiously, this is true also in centenarians, who have a GM enriched with Proteobacteria, which is made up also of “inflammatory” flora. These results suggest that the inflammatory or non-inflammatory role of certain bacteria can change based on different factors.

The effects of SCFAs can even be more striking, as it appears they can even modulate cancer cell proliferation in colon cancer, influencing overall tumor growth and response to drugs [94].

A short summary of the role of the various microbial species can be found in Table 1.

## 9. Microbiota and Vaccinations in the Elderly, a Love-Hate Relationship

Immunosenescence is considered the main reason behind the different response rates to vaccinations between older and younger patients, but it cannot account for other differences. An aspect that still is not completely understood is why some people exhibit strong immune responses to vaccinations, while others, who share similar characteristics in terms of age and health status, do not. Hepatitis B vaccination is one of the most studied vaccines in terms of efficacy: the CDC reports that response rates range from 80% to 95%, but there is a high variability already in persons over 40 years of age, which is hardly imputable to immunosenescence alone.

One of the most effective vaccines in the elderly population is influenza vaccine, which has been shown to be one of the most useful preventive measures in a longitudinal sense in people over 65 years of age, not only reducing the burden of related diseases in this group, but also improving cardiovascular health and reducing cardiovascular adverse events [98]. Yet, response to influenza vaccine in the elderly population can be as low as 20%, particularly due to immunosenescence [99]. Adjuvated-vaccines, high doses and recombinant vaccines have improved the response rates, but it still is not optimal [100,101]. In this picture, given its potential in producing “natural” adjuvants, such as LPS, GM could have a serious impact on this situation. Furthermore, microbiota helps differentiate B-lymphocytes: GM stimulates TLR-5 signaling and IL-1B and IL-6 production, all leading towards B-lymphocyte stimulation [102]. Another interesting aspect is that, when at an optimal status, GM produces SCFAs, which, again, have an important role in stimulating optimal B-lymphocyte metabolism and growth. Interestingly, also circulating levels of specific IgAs are higher in those whose GM produces high quantities of SCFAs.

Furthermore, the microbiota could have a role as a natural adjuvant for the response to influenza vaccine [68,103]. This is what emerged from a study which showed that TLR5-deficient mice, a molecule normally produced by GM, exhibit an altered response to the vaccine due to the altered mediated detection of the flagellin [104]. Normal antibody response was restored by oral reconstruction of the microbiota with a flagellated strain of Escherichia coli in mice previously treated with antibiotics or germ-free mice [104].

Another interesting perspective on the interactions between flu vaccination and microbiota has been provided by Bartley et al. [105]. It is a truth universally acknowledged that caloric restriction is useful in delaying the ageing process: Bartley and her colleagues suggest that this might take place through GM modulation. Another interesting aspect emerging from the study is the fact that flu vaccination is in itself a modulator of GM, even though not particularly potent.

While B-lymphocytes are the main agent in determining the response to vaccines, there needs to be a perfect equilibrium between all the parts of the immune system, for there to be an optimal vaccine-response. GM helps maintain healthy T-lymphocytes, stimulating specific CD8+ differentiation at the surface of the intestine, which has been shown to improve the response to infections [106].

Unfortunately, studies thus far have involved only small samples and results still need further validation, but the preliminary results suggest that there may be a place for microbiota-driven vaccinations. In particular, the use of probiotics has the potential of modifying the GM in a way that it could work as a natural adjuvant [107,108]. The use of prebiotics, as well, has been analyzed, even though it did not provide significant benefits in those who took them, when compared to those who did not. Yet, the explanation could be that, immunosenescence cannot be completely reverted, thus those who would benefit more from this kind of treatment, are probably those whose response is weaker [109].

## 10. Conclusions

Even though data on the interactions between microbiota and vaccination is still preliminary, the background information that is available strongly suggests that the immune modulation operated by the microbiota strongly influences vaccination response.

The interaction and interplay between vaccination response and microbiota is clearly bidirectional. As discussed above, vaccinations act as weak immune modulators of the GM, mildly increasing inflammation. On the other hand, GM is a strong modulator of inflammation: the role it has in inducing or reducing inflammation seems likely to have a serious effect on vaccinations in the elderly. The presence of a low-grade chronic inflammation is a key component in immunosenescence and creates an altered and not-as-effective reaction to vaccination. Overall, it is likely that GM is the strongest agent in the interplay with vaccinations.

Moreover, the elderly population is one if the ones that mostly benefit from vaccinations, but at the same time has a poorer response to this measure [70].

The possibility of modulating immune response in the elderly through microbiota manipulation (which includes dietary interventions, probiotics and reasonable antibiotic use) holds a lot of promise, in terms of improving effectiveness of vaccination protocols in the elderly.

## Figures and Tables

**Table 1:** Microbiota in the elderly.

Microbial Species	Effect	Reference
**Actinobacteria**	Improves response to oral vaccination	[56,59]
**Proteobacteria**	Low cellular and humoral response to vaccination, very common in centenerians	[56]
**Firmicutes**	Improves response to oral vaccination; most common in the “oldest old”	[57]
**Bacteroidetes**	Low humoral response to oral vaccination	[58]
***Enterococcus faecalis***	Inflammatory effect through ROS production; increases risk of epithelial damage	[67]
***Clostridium septicum***	Inflammatory effect; increases risk of infectious complications.	[67,73]
***B. fragilis***	Stimulates T-reg differentiation	[67]
***B. fragilis enterotoxigenic***	Stimulates Th-17 differentiation	[67]
***Bifidobacter spp.***	Promotes gut homeostasis through competition with pathogens; anti-inflammatory effects.	[85]
